# hg19KIndel: ethnicity normalized human reference genome

**DOI:** 10.1186/s12864-019-5854-3

**Published:** 2019-06-06

**Authors:** Harsh G. Shukla, Pushpinder Singh Bawa, Subhashini Srinivasan

**Affiliations:** 1 Institute of Bioinformatics and Applied Biotechnology, Biotech Park, Electronic City Phase I, Bangalore, 560100 India; 2 Manipal Academy of Higher Education (MAHE), Manipal, India

**Keywords:** Human reference genome, Disease predisposition, Population study, Variant calling, Major and minor alleles

## Abstract

**Background:**

The most widely used human genome reference assembly hg19 harbors minor alleles at 2.18 million positions as revealed by 1000 Genome Phase 3 dataset. Although this is less than 2% of the 89 million variants reported, it has been shown that the minor alleles can result in 30% false positives in individual genomes, thus misleading and burdening downstream interpretation. More alarming is the fact that, significant percentage of variants that are homozygous recessive for these minor alleles, with potential disease implications, are masked from reporting.

**Results:**

We have demonstrated that the false positives (FP) and false negatives (FN) can be corrected for by simply replacing nucleotides at the minor allele positions in hg19 with corresponding major allele. Here, we have effectively replaced 2.18 million minor alleles Single Nucleotide Polymorphism (SNPs), Insertion and Deletions (INDELs), Multiple Nucleotide Polymorphism (MNPs) in hg19 with the corresponding major alleles to create an ethnically normalized reference genome called hg19KIndel. In doing so, hg19KIndel has both corrected for sequencing errors acknowledged to be present in hg19 and has improved read alignment near the minor alleles in hg19.

**Conclusion:**

We have created and made available a new version human reference genome called hg19KIndel. It has been shown that variant calling using hg19KIndel, significantly reduces false positives calls, which in-turn reduces the burden from downstream analysis and validation. It also improved false negative variants call, which means that the variants which were getting missed due to the presence of minor alleles in hg19, will now be called using hg19KIndel. Using hg19KIndel, one even gets a better mapping percentage when compared to currently available human reference genome. hg19KIndel reference genome and its auxiliary datasets are available at 10.5281/zenodo.2638113

## Background

The first human genome sequenced and released in 2003 is a giant step in deciphering disease biology. One of the major goals of this effort, popularly known as The Human Genome Project, was to decipher the human proteome there by allowing for cataloging of all potential drug targets. This was also the first effort to provide a complete and accurate order of the 3 billion DNA base pairs that make up the human genome [1, 2]. Through several rounds of filling the gaps and improvements in the assembly, the hg19 version has become a stable assembly for more than a decade. As genomes of more and more individuals are sequenced, hg19 assembly became a de facto reference human genome.

A reference genome of an organism has multiple utilities. On the one hand, it provides a uniform coordinate frame enabling sharing/comparing of disease-, population- or individual-specific variations within scientific community. On the other, it can be used to flag out disease-specific variation in individual genomes. The assembly hg19 is currently widely used as a reference genome in our pursuit for mutations that causes/predisposes one to various diseases; thus, kick starting an era of personalized genomics or consumer genetics. The use of hg19 assembly as a human reference genome in disease biology, demands that hg19 carry non-pathogenic allele at all 3 billion positions by virtue of it being common to majority of individuals. This would help flag minor variations in individual genomes by comparing with the reference. However, it is well established that hg19 does not carry major alleles or non-pathogenic allele at all positions [3–5].

Currently hg19 provides a placeholder allele at each position on the chromosome so that major allele at each position can now be determined by sequencing/comparing large number of individuals from diverse population across the world. The 1000 genome project, which was launched to catalogue all variations in human by sequencing ~ 3000 strategically selected individuals to represent ethnic diversity globally. According to 1000 Genome Phase-3 dataset (Phase-3) [6] at more than 2 million positions hg19 harbors a minor allele and that the impact of these minor alleles on variant calls from individual genomes is severe [7]. For example, in this report it has been shown that 30% of individual variations fall within these positions and as large as 8% of the real variations in these minor alleles are missed if an individual is homozygous to the minor allele in hg19, with potential disease implications. Despite this, hg19 has been used for annotation of genome variations from millions of human samples. For example, Catalogue of Somatic Mutations in Cancer (COSMIC) database with around 50,000 potential cancer-specific variations [8] and ClinVar with 65,729 variations of clinical significance [9] all have been annotated with hg19 and can be expected to be peppered with false positives and negatives. Reports of false annotations in these databases are slowly emerging and are cumulative.

Recent studies have very aptly highlighted the issues that arise due to presence of a rare minor allele in the reference as well as provided tools to address them [3, 10]. Taking an example as discussed in *Barbitoff* et al [3] regarding Bardet–Biedl syndrome, a study done in pre-genomics era had identified the allele G as one of the pathogenic third allele according to the triallelic hypothesis [11]. This study was based on good old approach of making pedigrees analysis and sanger sequencing of the identified locus. A recent study misclassified the variant at that particular position, NM_031885.3:c.209G > A(C > T) (rs4784677), as a false positive [12]. Since the reference had the potential pathogenic allele C (G on opposite strand) and almost everyone had the wild type allele T (A on opposite strand), the authors concluded that this mutation (C- > T) cannot be deleterious. Here, the general assumption is that the reference allele is not pathogenic. In the individuals who are homozygous to the possibly deleterious reference C (G on opposite strand) allele as found in hg19, will be missed by the variant calling methods because it is not a variant (false negatives). Even today when we look at that position in ClinVar there is a conflicting clinical significance where the older submission states it is pathogenic while the new one suggests it is benign.

The other impact of using hg19 as reference is the compromised quality of read mapping stemming from minor alleles peppered across the length of the genome. Algorithms used for read mapping are designed to find identical stretches of genome that matches a given short read with 100% identity. At the expense of computation mapping tools have been implemented to accommodate minimal 1 or at the most 2 mismatches within the length of the reads while mapping so that variations between reads from individual genomes and the reference genome can be detected. The burden of large number of minor alleles in the reference, which are guaranteed to be resulting as mismatch for majority of individuals, will falsely be used as mutations within reads, confusing read mapping when a real variant is present in the neighborhood of minor alleles; thus essentially rejecting an authentic alignment or by picking another wrong loci as best match for a rejected read.

One extreme view is that the reference human genomes cannot be represented by linear string of ATGCs; only genomes of individuals can be represented accurately as linear strings. The most commonly used representation for non-linear sequence of data is graph representation [13, 14]. The advantage of graph representation of human genome is that one can include population specific information to improve accuracy of genome analyses. Large scale sequencing efforts like 1000 Genome Project have made it possible to represent human genome as graph genome by using population specific information. The question now is how does one map the reads of lengths say 100 bases to the graph reference genome? FORGe, which utilizes population genetics data to improve accuracy of genome alignment and variant calling offers an interim solution [15]. The model uses variants at every position in the reference by using information from population genetics. This model helps in prioritizing variants, which has relatively low fractions; thereby reducing reference bias and improving read alignment. A bigger challenge in migration to graph-based reference genome in favor of linear reference genome is the burden of developing necessary data science and technology.

The simplest way to correct for the minor alleles in hg19 is to create an ethnically normalized linear reference by replacing all the minor alleles in hg19 with the major alleles found in Phase-3. This allows continued use of existing tools/methodology, reducing the burden of false positives/negatives from downstream analysis, and improving variant calls by improving alignment. The advantages of having major alleles at all the position in the reference genome have been reported almost a decade ago [16]. In this report they have shown that the use of the major allele reference sequence results in improved genotype accuracy for disease-associated variant loci using information from family inheritance. There have also been reports of generating a consensus Korean reference genome [17]. Most of the previous studies published on the idea of creating a major allele reference genome have majorly focused on a particular ethnicity. Our group has reported the impact of minor alleles in hg19 by replacing only minor SNPs in hg19 with major alleles from population level information from multiple ethnicities from 1000 genome dataset to create a major allele reference genome (hg19K), so that like hg19, hg19K can also be used as a universal reference genome. But hg19K was limited to correcting only for minor SNPs in hg19 [7].

Here in this paper, we offer an ethnically normalized reference genome, hg19KIndel, where hg19 is replaced with major alleles of types including SNPs, minor INDELs and sequencing errors with the respective alleles from Phase-3 dataset. The main aim is to offer a reference genome that eliminates the burden of false positive and false negatives stemming from minor alleles in hg19 and offers improved alignment of reads.

## Results

### Creation of ethnically normalized genome: hg19KIndel

**Fig. 1:**
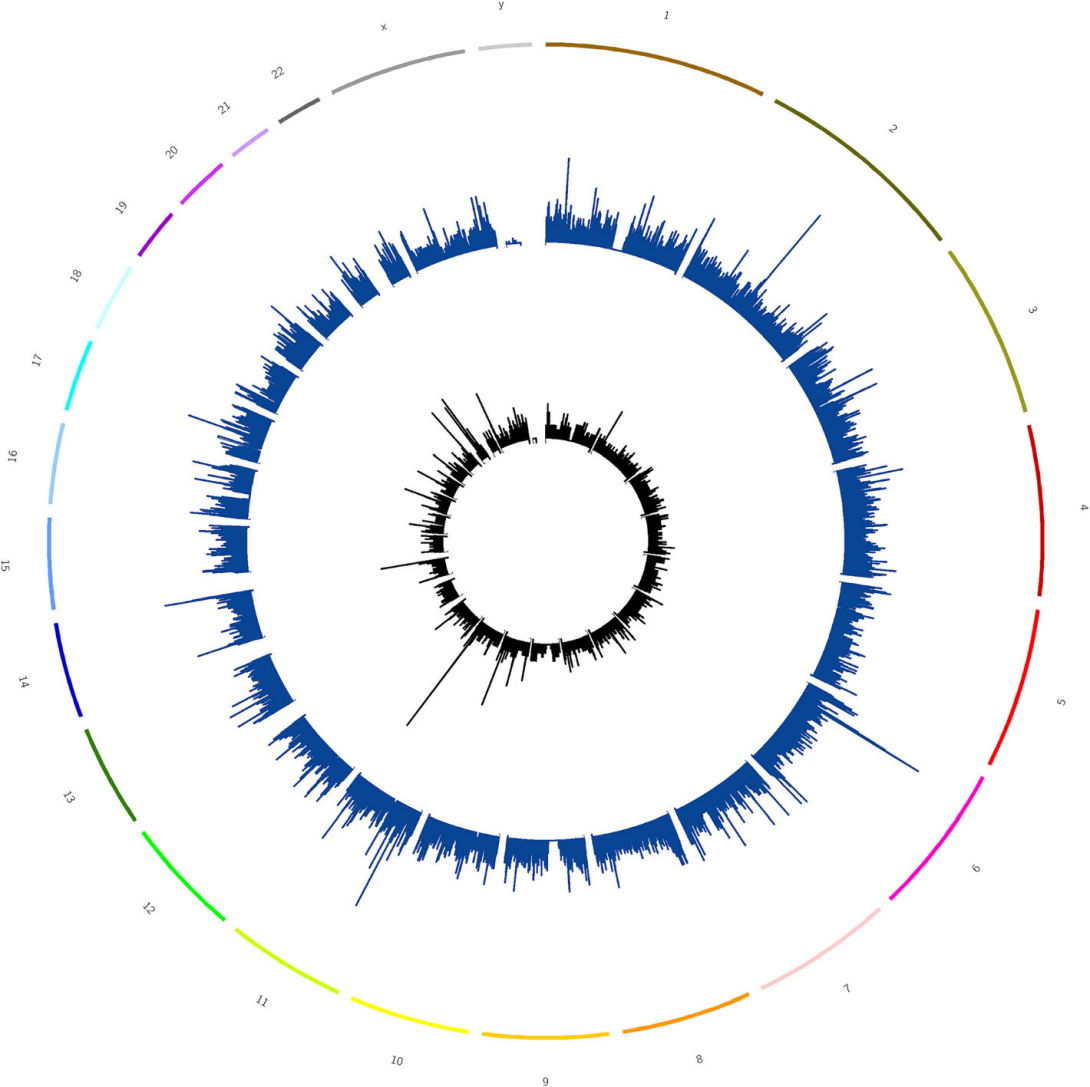
Distribution of SNPs (blue) and INDELs (black) replaced in hg19KIndel across all the chromosome

As per the 1000 Genome phase-3 dataset (Phase-3), there are around 81.3 million SNPs, 3.29 million INDELs and ~ 60 thousand other variants including MNPs and structural variants when compared to hg19. For each class of these variants, positions having alternate allele frequency (AF) > 0.5 were considered. There are 1.88 million SNPs, ~ 300 thousand INDELs and 3936 other variants with an alternate allele frequency > 0.5 (Table 1). In other words, hg19 harbors a minor allele at these positions compared to the ethnically diverse individuals included in the phase-3 dataset. Figure 1 shows the distribution of SNPs and INDELs across all the human chromosomes. As it can be seen, these positions are uniformly distributed across all the chromosomes. The outer circle in the plot represents all human chromosomes. The circle in blue represents 1.88 million SNPs that were replaced in hg19KIndel/hg19K and the innermost black circle represents the ~ 300 thousand INDELs replaced only in hg19KIndel.

**Table 1:** Number of variants called by 1000 genome project using hg19 as reference

Class	Total number of variants in 1000 genome phase-3 dataset w.r.t hg19		Filtered and replaced in hg19 (Alternate AF > 0.5in phase-31,000 genome dataset)	
	Total	Multi-allelic	Total	Multi-allelic
SNP	81,377,202	274,425	1,888,578	16,487
INDEL	3,299,133	103,598	305,634	14,732
SNP, INDEL	65,871	65,871	3401	3401
MNP	123	0	2	0
SV	59,551	0	535	0

Since the aim of 1000 genome sequencing project was to catalog variants from ethnically diverse population the major alleles in this database represent ethnically normalized non-pathogenic alleles at the respective positions. We have created a reference genome, hg19KIndel, that replaces the alleles at these positions in hg19 with the major alleles from the Phase-3 dataset. While majority of the variants were biallelic, significant number (Table 1 columns 3 and 5) of them were multi-allele challenging the substitution process. For positions with multi-alleles the major alleles out of all the alternate alleles were used to replace the respective allele in hg19. Also, there were certain positions where both INDELs and SNPs are present (Table 1 Row 4). In such cases the allele that had the highest allele frequency was used for replacement.

Column 2 and 3 shows the number of variants called in 1000 Genome phase-3 dataset with respect to hg19. Columns 4 and 5 shows the number of variants that have an alternate allele frequency of greater than 0.5, representing the minor allele position in hg19.

Replacing SNPs in hg19 with major alleles from phase-3 dataset is relatively straight forward as this introduces no change in coordinate frame [7]. The challenge is in replacing INDELs because the coordinates of INDELs will keep changing dynamically during replacement. Once an INDEL is replaced the subsequent positions downstream to it must be changed accordingly. In other words, replacing INDELs involves shifting coordinate frames contiguously until all changes are introduced; demanding a log of changes to be maintained to restore coordinates. To both validate and for the reference to be useful to larger scientific community, there is a need for databases compatible with hg19KIndel. These include creating chain formatted files routinely used by UCSC to liftover information from one assembly to the other and variant annotation database compatible with the version of the assembly.

### Creating chain file for liftover: hg19KIndel2hg19

Every genome assembly has unique coordinate frames introduced either by the additional sequencing of missing/gap regions or due to unique frameshift mutations and other structural variations introduced along the chromosomes. Thus, variants that are exactly at the same position relative to a gene changing the same amino acids may have a different coordinate from different assemblies; making it clumsy to compare variants predicted from different versions of assemblies. For this reason, a liftover tool is made available by University of California at Santa Cruz (UCSC) as part of genome resources. To enable liftover between various version of assemblies, UCSC also provides a chain file, which represents pairwise alignment blocks between any two assemblies. Chain files are generated by doing a whole genome alignment between the two assemblies to find syntenic blocks so that the coordinate liftover can be performed. For the purpose of liftover, hg19KIndel is treated as a yet another version of human genome assembly.

A chain file *hg19KIndel2hg19* was generated in-house to enable liftover of variants called using hg19KIndel to the most widely used human reference genome assembly, hg19. To validate *hg19KIndel2hg19* we compared the variants called using hg19 and hg19KIndel using the same pipeline for a single dataset before and after liftover. Since even a single frameshift INDEL can shift the positions of all variants following the INDEL, one can expect near-null variants that will be called at identical coordinates between the two calls despite use of the same dataset in both cases. However, a proper liftover of the variants from hg19KIndel to hg19 should render most of the variants from hg19KIndel to the same coordinate frame as hg19. Thus, after liftover one can expect a large percentage of overlap between the two calls; thus, authenticating both the chain file and hg19KIndel genome. As shown in Table 2, lack of common SNPs before liftover (Column 5) and majority SNPs common after liftover (Column 6), validates the *hg19KIndel2hg19* chain file created here.

**Table 2:** Comparison of variants called using hg19 and hg19KIndel before and after lifting over the coordinate of variants

Sample	Variant class	Total Calls (hg19)	Total Calls (hg19KIndel)	Overlap	
				Before Liftover	After Liftover
In house whole exome sequencing sample (WES)	SNP	45,285	33,676	34	30,563 (68%)
	INDEL	4741	3494	9	3090 (65%)

Although ~ 99.85% of the variants called using hg19KIndel are lifted over to hg19 successfully, the lower number (68%) of overlap compared to hg19 variants is expected as a result of 25% false positives inherent in hg19-based prediction, which are expected to be missing in hg19KIndel by design. Furthermore the 91% overlap compared to hg19KIndel calls results from false negatives in the hg19-based calls that are picked up only by hg19KIndel. There is a small number of variants that are false negative in hg19-based calls but are present in hg19KIndel-based calls because of improved alignment offered by hg19KIndel not because of minor allele.

### hg19KIndel compatible annotation file: refGeneKINDEL

Considering that even a single INDEL in the chromosomes could cause shift in the start and end coordinates of all the exons and introns of coding genes, one requires a gene annotation file compatible with each assembly. refGene file from UCSC is one such annotation database which contains start and end information of exons and introns of all the transcripts for a given assembly along with the frame information in which each exon is translated. The complexity in creating refGene is that exons from a single gene can translate in different reading frames to stitch a full-length protein. The reading frames themselves can change depending on errors in the exons preceding it. A wrongly annotated refGene file will have consequences in assigning impact for a given mutation within coding regions. For this reason, the frame information is carefully curated in refSeq file provided by UCSC, handling even cases when hg19 reference sequence is not totally compatible with refSeq mRNA sequences i.e. when there is an insertion or deletion in hg19 w.r.t to mRNAs. SnpEff uses this frame information for translation of sequences extracted from genome.

**Fig. 2:**
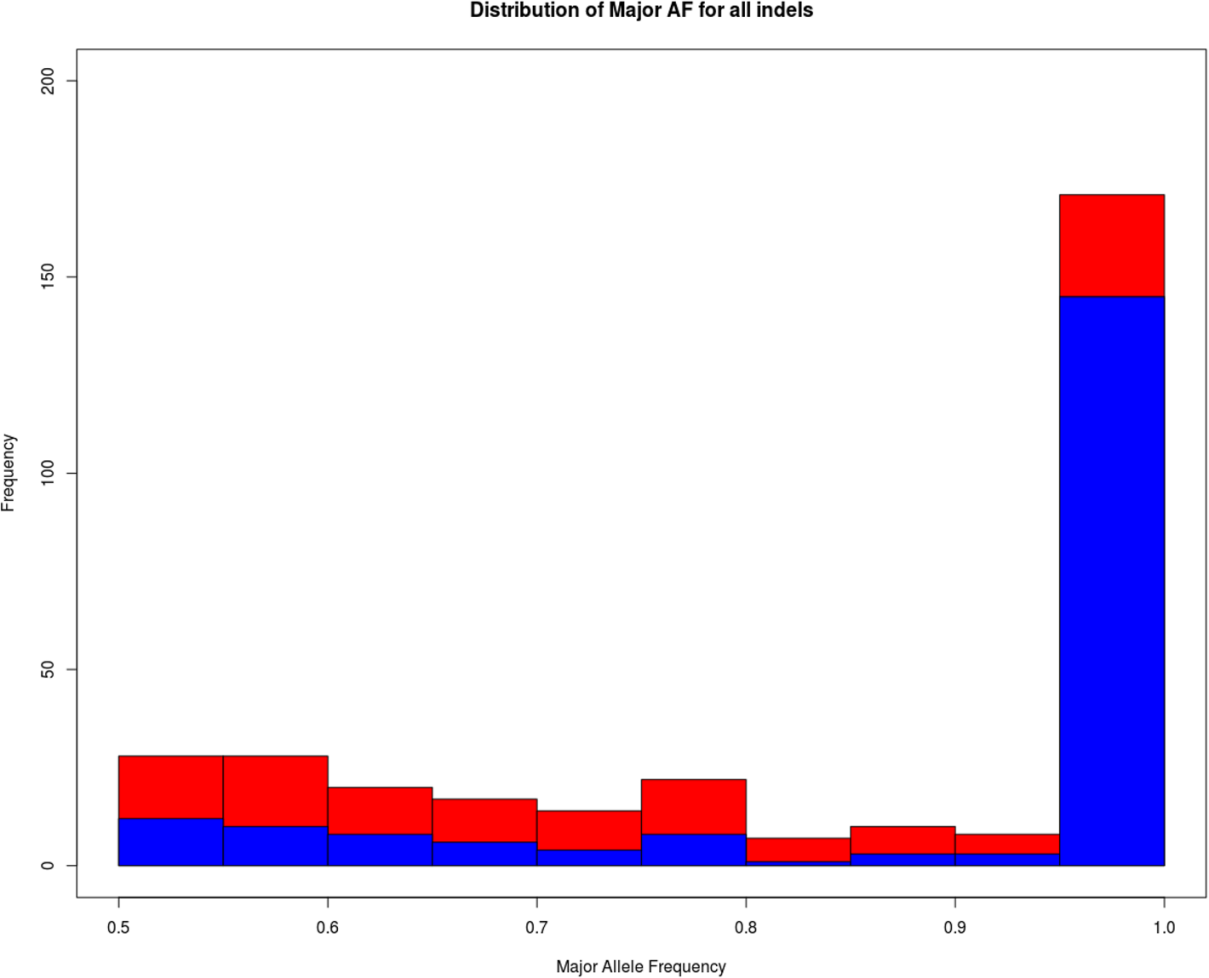
Distribution of major allele INDELs having alternate allele frequency greater than 0.5 that are in-frame (red) and out-of-frame (blue) falling within the coding exons. Out of the frame INDELs (blue) is plotted over inframe INDELs (red)

Due to incompatibility of coordinates between hg19KIndel with any other existing assemblies, there is a need for creating hg19KIndel compatible refGene file. Only just lifting the entire hg19 refGene to hg19KIndel coordinate frame will not suffice, because the sequence extracted from the genome using the annotation finally represents a functional protein sequence. To build a correct annotation for hg19KIndel, the distribution of major INDELs that were replaced had to be tracked, especially the ones occurring in exonic regions because these INDELs are the one that will likely affect the protein being translated. These INDELs can be both frameshift as well as non-frameshift INDELs. Figure 2 shows the distribution of major INDELs having alternate allele frequency greater than 0.5 and fall within the CDS regions of exons (excluding the UTR regions).

The red bars indicate the distribution of in-frame INDELs whereas the blue bars indicate the distribution of out-of-frame INDELs. This graph shows that at almost all allele frequencies the number of out-of-frame INDELs is comparatively lower than the in-frame INDELs, which is expected. However, at the allele frequency of 0.9–1.0 a majority of the INDELS are out-of-frame. These are likely errors in hg19 assembly, which were caught by 1000 Genome project. Others have reported such errors as well. For example, Lin et al. [18] says that most of the mis-annotations on the human reference genome come from the frameshift INDELs where the homozygous allele frequency is 100%. They found out that several reference genome annotations might need to be updated due to the high prevalence of these homozygous frameshift INDELs. According to this report, high frequency homozygous INDELs should be the point of interest for updates in human reference genome keeping in line with suggestion by the structural variant studies [19].

Since hg19KIndel had these suggested updates the annotation also must capture the same. Consequently, after lifting over coordinates another step of error correction had to be done to check if the frame information is consistent with hg19 annotation and updating the frame wherever necessary. In some cases, adding to the complexity, the error lead to change in the total number of exons in a given gene. For example, if there was a small insertion (2-8 bp) in hg19 (w.r.t to mRNA) it was previously classified as intron. During error correction these small introns were removed, and two neighboring exons were merged and there by updating the frame information. Similarly, if there was a deletion in hg19 causing breaking of an exon into two, such positions were also corrected, and the frame information was adjusted accordingly. All such cases were carefully assessed and corrected as required while deriving the annotation for hg19KIndel to create refGeneKINDEL file. Even after this careful examination, for a few genes out-of-frame INDELs could not be fixed.

### Compatible variant annotation database: snpEffectPredictor_hg19KIndel

After variant calling, there is a need to understand their functional impacts. As hg19KIndel is a different genome assembly from hg19, we cannot directly use the existing SnpEff [20] databases for annotation. We have developed a database compatible to hg19KIndel, which can be used by SnpEff for annotation of variants. One can use the logic of liftover and can technically just lift the entire annotation of hg19KIndel to hg19 coordinate frame. But lifting the coordinates is not enough as the reference genome hg19KIndel carries the corrected major alleles which are different when compared to hg19. Functional annotation of variants called by using hg19KIndel is expected to report different reference allele than those from hg19 at these positions even after liftover. Therefore, creation of a new SnpEff database compatible with hg19KIndel was necessary. Using refGeneKINDEL and hg19KIndel genome we built a SnpEffKINDEL database.

### Validation of hg19KIndel, refGeneKINDEL, chainFileKINDEL and snpEffKINDEL databases

While building a SnpEff annotation database, it performs sanity check to authenticate the genome and the companion databases. During sanity check, sequence of every transcript in the annotation file is extracted from the reference genome using the gene structure defined in the annotation file. These extracted transcript sequences are matched against their respective transcript sequence present in reference transcript database (refSeq). At the protein level, using the gene sequence extracted from the genome plus the frame information in the refGene file the CDS are translated into corresponding proteins and then compared to those from refSeq, which is also given as input to the tool. In other words, sanity check gives the percentage of match between the predicted CDS from hg19KIndel and those from refSeq database. We performed sanity check for all three genomes hg19, hg19K and hg19KIndel against the ~ 65,000 transcripts in the refSeq database.

To create a baseline for sanity check, SnpEff database was built for hg19 using hg19 and the corresponding refGene annotation file downloaded from UCSC. According to this, 92.81 and 95.2% of genes had exact matches to those from refSeq at the transcripts and protein level respectively (Table 3). For building SnpEff database for hg19K, which differs from hg19 at all minor allele SNP positions, while still conforming to the hg19 coordinate frame [7], the same hg19 refGene file along with the genome hg19K is used. The percentage match at transcript and protein levels drops to 70.48 and 85.79% respectively. While this drop is large, it can be accounted for by the change of minor allele SNPs by major alleles in hg19 within the genes. For sanity check of hg19KIndel genome using refGeneKindel annotation file both generated in-house, the match percentage between refSeq and hg19KIndel only dropped slightly lower (~ 0.5%) at transcript level and even smaller (~ 0.04%) at the protein level (Table 3) compared to those with hg19K. Considering that the number of minor INDELs/errors corrected in hg19KIndel is a small fraction on top of the number of minor SNPs replaced in hg19K, one should expect only a small drop in match between the hg19K and hg19KIndel. The high percentage (85%) of match between protein sequences derived from hg19KIndel using refGeneKINDEL and refSeq offers a test of compatibility between hg19KIndel and refGeneKINDEL and thus, offering validation for both.

**Table 3:** SnpEff sanity check of hg19, hg19K and hg19KIndel

Sanity Check while building compatible snpEff database	Genome	Compatible annotation database	Reference mRNA	Match percentage between the respective genome and refSeq	
				Protein level	mRNA level
Existing baseline	hg19	refGene	refSeq	92.81	95.2
After replacing only minor SNP alleles with major alleles in hg19	hg19K	refGene	refSeq	85.79	70.48
After to replacing minor INDELS with major alleles in hg19K	hg19KIndel	refGeneKINDEL	refSeq	85.75	69.96

### Assessing extent of correction of false positives/negatives by hg19KIndel

Three variant files were generated using hg19, hg19K and hg19KIndel assemblies for two whole genome and whole exome datasets including NA12878 (ERR194147) from Caucasian and another dataset sequenced in house from individuals with Indian ethnicity. All the variants from hg19KIndel were lifted over to hg19 coordinates to enable comparison. Table 4 represents the comparison made between the variants called on both the samples using assemblies hg19, hg19K and hg19KIndel for whole genome datasets. Variants from both the samples were concordant in terms of false positives (FP) and false negatives (FN) for both SNPs and INDELs for both whole genome (Table 4) and exome data (Table 5). Comparison of hg19 and hg19KIndel a rate of ~ 30% FP and ~ 8% FN was observed for SNPs; and a rate of ~ 30% FP and ~ 12% FN was observed for INDELs. A similar percentage of FP and FN (for SNPs) was observed during our earlier efforts of creating hg19K. The above observations provide evidence that even when individuals from two different ethnicities and two different sequencing strategies are used the same rates of false positives and negatives are observed thereby confirming our assumption - hg19KIndel is truly a normalized representation of human reference genome.

**Table 4:** Variant comparison for WGS samples across three different versions of human genome

Sample	Genome		Variant class	Total Calls I	Total Calls II	Variant count			% false positives in I	% false negatives in I
	I	II				common	Unique to I	Unique to II		
NA12878	hg19	hg19K	SNP	3,691,042	2,859,214	2,624,382	1,066,660	23,482	28.89	8.21
			Indel	809,628	807,394	789,612	20,014	17,782	2.47	2.20
	hg19	hg19KIndel	SNP	3,691,042	2,852,155	2,611,050	1,079,992	241,105	29.26	8.46
			Indel	809,628	651,931	570,962	238,666	80,969	29.48	12.53
	hg19K	hg19KIndel	SNP	2,859,214	2,852,155	2,837,316	21,898	14,839	0.76	0.52
			Indel	807,394	651,931	582,100	225,294	69,831	27.90	10.80
In house Whole genome sample	hg19	hg19K	SNP	3,652,081	2,788,319	2,551,695	1,100,385	236,623	30.13	8.48
			Indel	665,692	664,663	650,704	14,988	13,959	2.25	2.10
	hg19	hg19KIndel	SNP	3,652,081	2,783,025	2,541,407	1,110,674	241,618	30.41	8.68
			Indel	665,692	528,233	461,953	203,739	66,280	30.60	12.63
	hg19K	hg19KIndel	SNP	2,788,319	2,783,025	2,768,869	19,448	14,156	0.70	0.50
			Indel	664,663	528,233	470,655	194,009	57,578	29.19	10.97

**Table 5:** Variant comparison for whole exome sequencing samples across three different versions of human genome

Sample	Genome		Variant class	Total Calls I	Total Calls II	Variant count			% false positives in I	% false negatives in I
	I	II				common	Unique to I	Unique to II		
NA12878	hg19	hg19K	SNP	43,313	33,064	30,046	13,267	3018	30.630	9.127
			Indel	4251	4245	4191	60	54	1.411	1.272
	hg19	hg19KIndel	SNP	43,313	33,023	29,970	13,343	3053	30.803	9.245
			Indel	4251	3202	2827	1424	375	33.498	11.788
	hg19K	hg19KIndel	SNP	33,064	33,023	32,967	97	56	0.293	0.169
			Indel	4245	3202	2863	1382	339	32.555	10.657
In house whole exome data	hg19	hg19K	SNP	45,285	33,700	30,633	14,652	3067	32.35	9.10
			Indel	4741	4740	4680	61	60	1.28	1.26
	hg19	hg19KIndel	SNP	45,285	33,673	30,563	14,722	3110	32.50	9.24
			Indel	4741	3494	3090	1651	404	34.82	11.65
	hg19K	hg19KIndel	SNP	33,700	33,673	33,602	98	71	0.29	0.21
			Indel	4740	3494	3122	1618	372	34.13	10.73

Table 5 shows that for NA12878 exome sample FP rate of ~ 31% and FN ~ 10% for SNPs; ~ 33% FP and FN ~ 11% for INDELs. Due to the complexity involved in library preparation of the exome datasets it is subject to many more biases and thus the of numbers called (FP and FN) we obtained are little more variable. Additionally, the numbers of variants obtained from exome studies are much smaller (as compared to Whole genomes). As a result, the percentage of false positive and negatives coming from exome sequencing data are subject to higher fluctuations depending on the dataset being analyzed.

The pairwise comparisons were made in order to make sure that the change in numbers for both SNPs and INDELs while lifting over from hg19 to hg19K to hg19KIndel conform to change in the underlying genome sequence. For example, while comparing hg19 and hg19K, both genomes only differ by base pair changes, we should expect only SNPs and not INDELs to be significantly different and that is what is observed (Table 4). Similarly, when we compare hg19K and hg19KIndel, since they differ only by INDELs very less unique SNPs and significant change in INDELs is expected and observed. Now when we compare hg19 and hg19KIndel with respect to SNPs most changes in the SNPs called comes from replacement of major allele SNPs and the numbers should match that of comparison of hg19 and hg19K for SNPs. Similarly, when we compare hg19 and hg19KIndel with respect to INDELs most changes in INDELs called comes from replacement of minor INDELs and the numbers should match that of comparison of h19K and hg19KIndel for INDELs. The numbers do agree with our expectations and provides enough proof that most variants being called differently during pairwise comparison do come from the changes we introduced in the genomes and are at the same positions. A small number of variants, like INDELs unique to hg19 and hg19K (comparison of hg19 and hg19K) and SNPs unique to hg19K and h19KINDEL (comparison of hg19K and hg19KIndel) perhaps come from improved alignment (discussed elsewhere) due to use of different references. Thereby a very small percentage of SNPs and INDELs called in hg19KIndel are expected to be at positions other than the ones we changed while creating hg19KIndel.

Not just the change in the number of variants, we also looked at the change in the effects of variants caused using hg19KIndel. To do so, we annotated variants from NA12878 sample using SnpEff. As shown in Table 6, the number of HIGH impact variants decreased from hg19 to hg19KIndel. This can be due to absence of frameshift variants, which were called due to errors in sequencing in hg19. All such positions have now been corrected in hg19KIndel, thereby reducing the downstream burden on variant discovery and validation. Here in order to do a fair comparison the SnpEff databases for both hg19 and hg19KIndel were built in-house.

**Table 6:** SnpEff comparison for exonic variants of NA12878 across the three human reference genome hg19 and hg19KIndel

	hg19	hg19KIndel
	Number of Variants	
SNP	43,321	33,030
INS	2247	1596
DEL	2086	1675
	Number of effects by Impacts	
HIGH	578	309
LOW	25,309	19,629
MODERATE	19,247	14,365
MODIFIER	125,507	95,814
	Number of effects by functional class	
MISSENSE	18,872	14,034
NONSENSE	83	77
SILENT	23,560	18,180

### Examples for correction of false positive by hg19KIndel

**Fig. 3:**
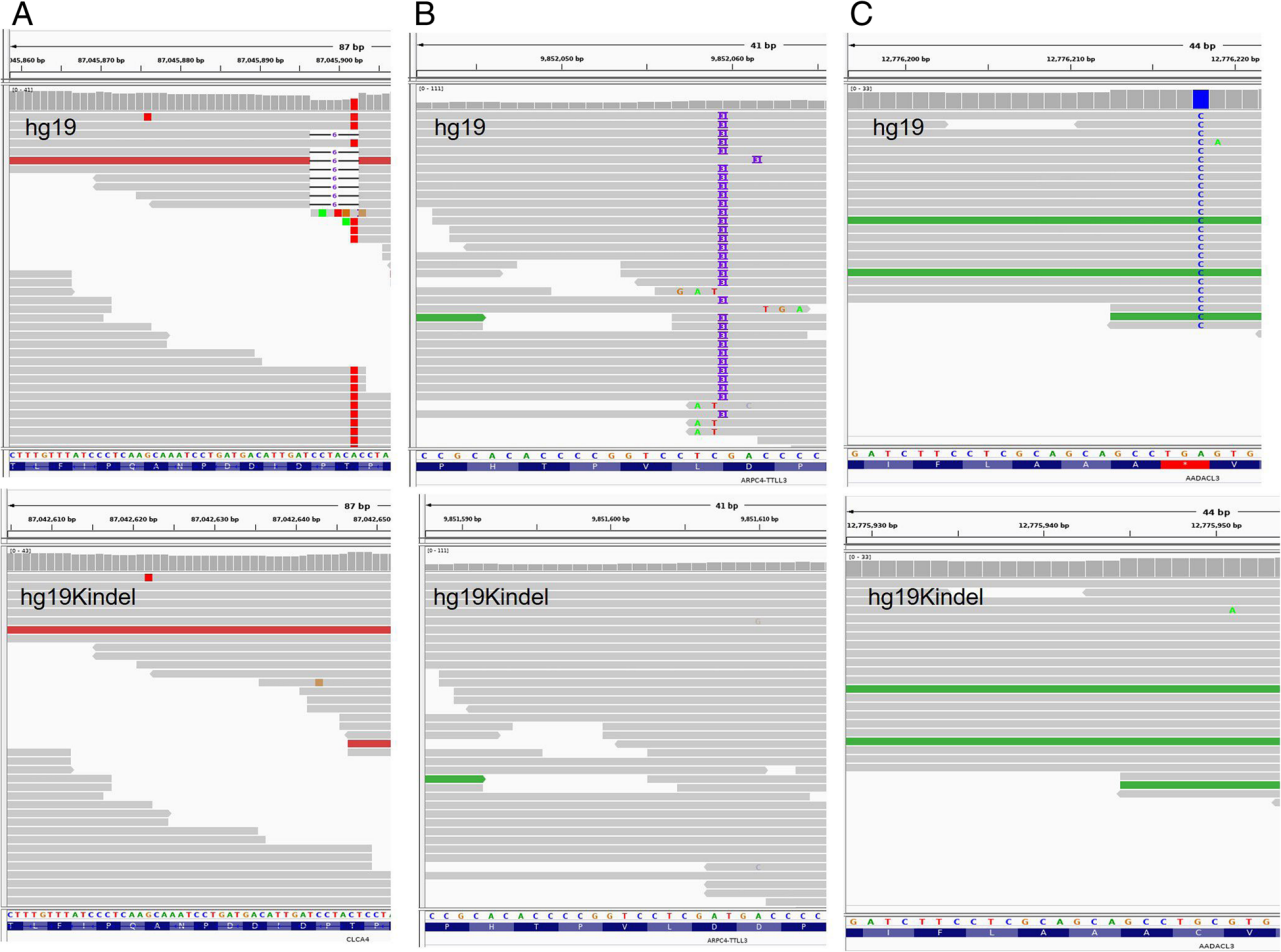
Examples of corrections in false positive variants called using hg19 and are corrected for in hg19KIndel. **a** Deletion in CLCA4 gene on chr1:87045896. **b** Insertion in ARPC4 gene on chr3:9852059. **c** SNP in AADACL3 gene on chr1:12776218

The three examples in Fig. 3 shows that hg19 based variant calls have picked up on a false positive with very high confidence, which is corrected in hg19KIndel based alignment. In Fig. 3a (top) a deletion mutation in CLCA4 gene on chr1:87045896, which is a minor allele but is reported as a mutation in an individual genome using hg19. It has now been corrected and is not called for in hg19KIndle based variant call (Fig. 3a, bottom). Likewise, in Fig. 3b (top) an insertion in ARPC4 gene on chr3:9852059, which is a minor allele is erroneously called as a variant in an individual genome when hg19 is used as a reference and is now not called in hg19KIndel based variant call (Fig. 3b, bottom). Similarly, Fig. 3c (top) gives an example of a SNP in AADACL3 gene on chr1:12776218. Here hg19 has a pre terminating stop codon due to presence of minor allele A meaning that hg19 probably contains the non-functional allele. But when a mutation is reported in hg19 from A to C it termed as stop lost and is classified to have a high impact. But when we replace A with corresponding major allele C the pre-terminating stop codon disappears from the annotation as well as from the sample when called using hg19KIndel (Fig. 3c, bottom). This is one of an example discussed in Table 6, which represents the drop in number of variants having overall high impact as we go from hg19 to hg19KIndel.

### Examples for correction of false negative by hg19KIndel

**Fig. 4:**
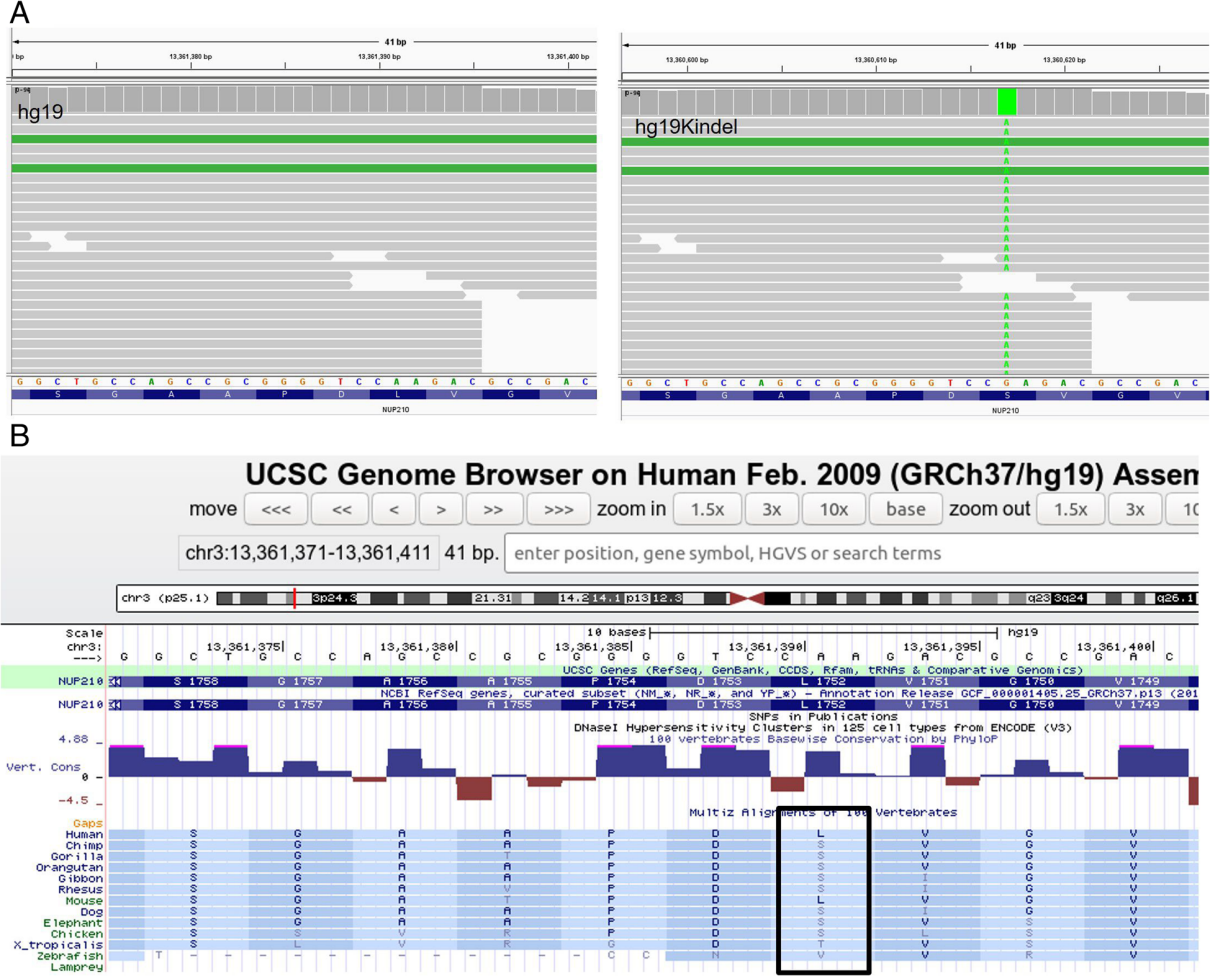
Example of a false negative variant call. **a** SNP on gene NUP210 at position chr3:13361391 cannot be detected while using hg19 as the allele reported at this position is the major allele. Using hg19KIndel the minor allele at this position can be detected. **b** UCSC snapshot of the position chr3:13361391 shows that Leucine is there at that position in humans whereas it is Serine which is conserved across other species. If we replace the allele with its major allele it will be Leucine in humans as well. We have corrected for such positions as well in hg19KIndel

Erroneous mapping due to non-availability of major alleles at all the positions in hg19 also leads to masking of some true variants. Such events are called false negatives. While false positives can be subtracted using 1000 genome phase 3 dataset, there is no way to restore false negative events from hg19-based variant calls. Figure 4 gives examples of such events. In Fig. 4a (left), hg19 reports a minor allele at position chr3:13361391 on NUP210 gene, so if an individual is homozygous to this minor allele, the mutation won’t even be called. On the other hand, if the individual harbors a major allele it will be inaccurately called as a variant leading to false positive. By replacing the position with major allele hg19KIndel (Fig. 4a, right) takes care of both false positive and false negative problems stemming from minor alleles in hg19. Figure 4b shows UCSC snapshot of the position chr3:13361391 in hg19 which shows that Leucine is coded for at that position in hg19 whereas it is highly conserved Serine across most other species (bottom panel highlighted by black). Interestingly, if one replaces the minor allele with its major form it will be Serine in human as well.

### Examples of correction in sequencing errors in hg19KIndel

**Fig. 5:**
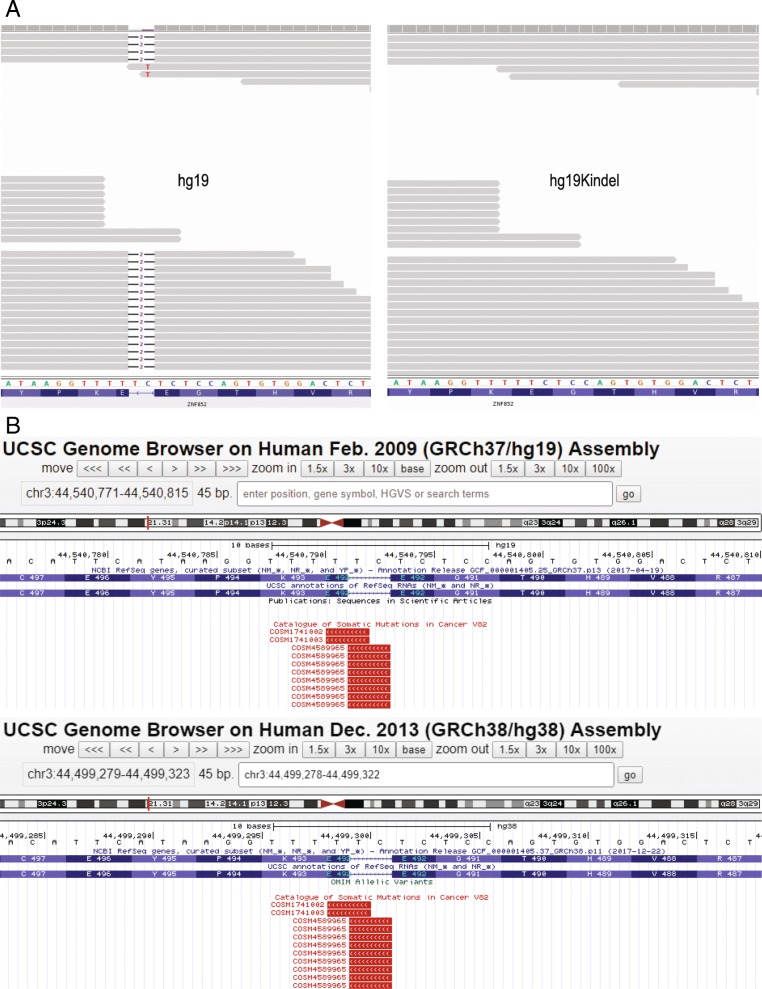
Examples of sequencing errors in hg19 that are corrected in hg19KIndel. **a** Deletion in ZNF852 on chr3:44540791 **b** This mutation is reported to be cancer specific by COSMIC in both hg19 and hg38 reference genome

Some of the major alleles in Phase-3 dataset have allele frequency as high as 1 or very close to 1. These variants likely represent sequencing errors in hg19 and therefore no matter which sample dataset is analyzed, these variants will be called in almost all samples. Correcting for these errors in reference will remove these spurious calls. During the release of hg38 in 2013, GRC used the data from 1000 Genome project phase-1 dataset to correct for some of these sequencing errors [13]. Since then Phase 3 dataset has identified many more such errors. Such sites are reported as “point of interests for human reference genome updates” [18]. Figure 5a (left) shows a deletion in the gene ZNF852 at chr3:44540791. This position is called as a variant when hg19 (Fig. 5a, left) is used as a reference genome but not while using hg19KIndel (Fig. 5a, right). This deletion is reported to be cancer specific by COSMIC database (COSM4589965) in both hg19 (Fig. 5b, top) and hg38 (Fig. 5b, bottom). Even hg38 is not corrected for this position whereas hg19KIndel has this corrected (Fig. 5a, right).

**Fig. 6:**
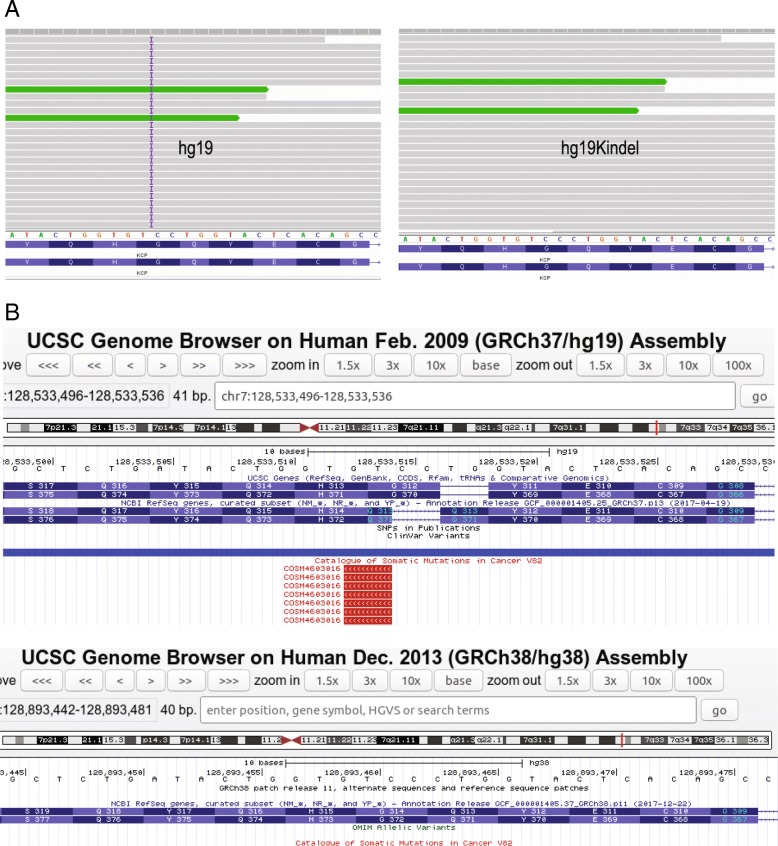
Examples of sequencing errors in hg19 that are corrected in hg19KIndel and in hg38. **a** Insertion in KCP gene on chr7:128533514. **b** UCSC screenshot showing the mutation in KCP gene reported by COSMIC while using hg19 but corrected for in hg38

Figure 6a (left) shows an insertion (frameshift) in gene KCP at chr7:128533514 which is called while using hg19, but is corrected in hg19KIndel (Fig. 6a, right). This insertion is reported to be cancer specific mutation by COSMIC (COSM5713146) but is a sequencing error and is wrongly annotated. Figure 6b a screenshot showing COSMIC track from UCSC browser for this position in both hg19 (Fig. 6b, top) and hg38 (Fig. 6b, bottom). This position is corrected in hg38 (Fig. 6b, bottom) and in hg19KIndel based variant call (Fig. 6a, right). Also, if we look at RefSeq track in the UCSC image of hg38 (Fig. 6b, bottom) and gene annotation track in our hg19KIndel IGV image (Fig. 6a, right) we find that both the tracks are exactly same. This in a way provides further evidence that the steps we performed (liftover, validation and error correction) for generating hg19KIndel RefSeq file were in the right direction and the final RefSeq file we generated for hg19KIndel is quite accurate.

### Extent of improvement in read alignment in hg19KIndel

**Fig. 7:**
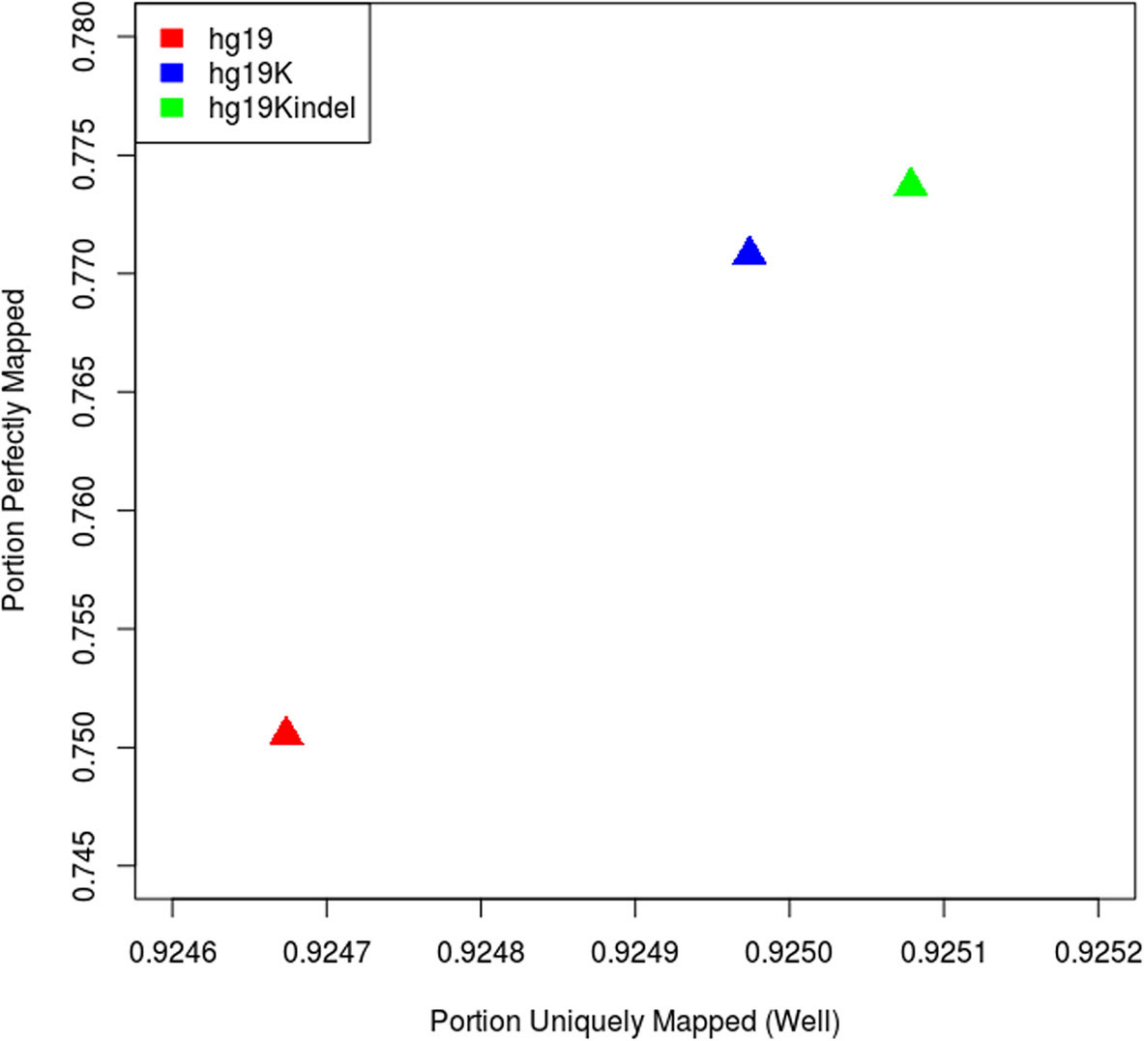
NA12878 was mapped in an unpaired way on all three genomes. This graph shows an improved in mapping when the sample is mapped onto hg19KIndel, in comparison to hg19 and hg19K

Most of the aligners are inherently biased towards mapping reads containing the reference allele. The position in reads containing the non-reference alleles in pure algorithmic perspective are considered as mismatches and they are penalized. This can be called as reference bias and because of this the reads significantly differing from the reference may not be mapped to their true positions. Reference bias can cause such reads to be unmapped in some cases or mapping to a totally wrong position in worst cases. Incorrect alignment of reads from individual genomes also leads to errors in downstream variant calling. This, however, can be mitigated by replacing all minor alleles by corresponding major alleles. To assess different representations of a reference, Novak et.al [21] introduced a method in which they plotted the portion of reads mapping perfectly and portion of reads mapping uniquely. Perfectly mapped reads are the one that exactly match the reference (no mismatches; no INDELs). Uniquely mapped reads are the ones that have only one primary alignment (no secondary alignment at all) or the reads do have secondary alignment, but the primary alignment score is significantly greater than the secondary alignment score. Here, NA12878 dataset was mapped to all three genomes (hg19, hg19K and hg19KIndel) in an unpaired fashion. Portion of reads mapping perfectly and portion of reads mapping uniquely where calculated and plotted for all three genomes. It can be seen in Fig. 7, when we go from hg19 to hg19K to hg19KIndel both the parameters are increasing proving that hg19KIndel is a more improved reference representation as compared to hg19. The total number of perfectly mapped reads increased to 609.11 million when hg19KIndel was used as a reference in comparison to hg19 (590.87 million) and hg19K (606.82 million). The number of uniquely mapped reads also went up to 728.28 million for hg19KIndel as compared to 727.96 million for hg19 and 728.19 million for hg19K.

For one of the samples, NA12878 we also analyzed variant calling on hg19K and looked at FP and FN (Tables 4 and 5). On comparison of hg19K and hg19KIndel (keep in mind - they differ by only major INDELs) ~ 0.6% unique SNPs were found. If the assumption that references bias is being mitigated as one goes from hg19 to hg19K to hg19KIndel is valid, these unique variants represents false positives and false negatives in a true sense. For example, position chr1:87045902 in Fig. 3a (top) there is a variant representing mutation of A to T (red color; False Positive). This variant arises because the reads near the minor allele locus are aligned incorrectly leading to an erroneous call. As hg19KIndel has been corrected for minor INDEls, it has helped in improving the variant called around these INDELs thereby further improving in variant calling for SNPs as well (Fig. 3a, bottom).

## Discussion

Here, we offer an ethnically normalized human genome, hg19KIndel, built by incorporating major alleles from phase-3 variants of 1000 Genome project into hg19. This was done in two phases. In hg19K [7] only SNPs were replaced with major alleles to evaluate the impact of minor alleles on variant calling. In the current version hg19KIndel all the major SNPs, major INDELs and sequencing errors are replaced in hg19 to provide full advantage of ethnically normalized major alleles in variant calling. Unlike hg19K, hg19KIndel has coordinate frame incompatible with hg19 requiring development of compatible tools and annotation databases for comparison with hg19-based variants. Here, we not only provide hg19KIndel genome for improved variant calling using existing pipelines but also provide hg19KIndelSnpEff database and *hg19KIndel2hg19* liftover tool for annotation and comparison respectively.

We have shown that hg19KIndel eliminates false positives and false negatives in variant calls from individual genomes, which has been shown to be as high as 30 and 8% respectively. While false positives in other applications are usually considered a minor nuisance and usually result from low significance, in this case false positives are of high significance as reads from these loci for most individuals are likely to have the major variant. Furthermore, annotation of hundreds of thousands of samples with hg19 resulted in too many false positives that has already proliferated the variant databases with mis annotation including COSMIC and ClinVar. A recent publication rightly pointed out that there is significant over prediction in ClinVar database [22].

False negatives can have grave consequences in population genetics and rare recessive disorders, where disease-specific alleles are expected to be homozygous to minor alleles in hg19 and is totally missed. In one instance our group reported that even the reference amino acid, glutamine, in hg19 was arginine based on hg19KIndel reference, which is conserved across all species. Meaning that glutamine in hg19 in that position could be deleterious but will not be reported for individuals homozygous to glutamine [7]. The other problem with false negatives is that for data from millions of samples that has already been analyzed it is lost cause unless someone re-analyzes those data.

All variant calling pipelines depend heavily on the sensitivity of the mapping tools that is used to align reads sequenced from individual genomes to the reference genome. The impact of minor alleles and errors in reference genomes also causes misalignment of reads derived from that region. Graph-based representation of human genome has been proposed. However, compromising the simplicity of linear genome will require abandoning existing tools/pipelines, sharing of variant information, and higher computational footprint. With genome data pouring every minute of the day, there is a need to have an improved genome that does not require wholesale change in tools and pipelines. hg19KIndel is the first effort to improve a version of the genome assembly horizontally to graduate it into a human reference genome.

The reference transcript database refSeq has one or more mismatch in as large as 7.19% of the genes with respect to hg19. This is even though much of refSeq mRNAs came from hg19. These mismatches from hg19 obviously must have come from collating mRNA sequences for refSeq from other sources derived from other individuals not used in the human genome effort. However, a larger jump in percentage of mismatch (29%) between refSeq and hg19K/ hg19KIndel is surprising and would suggest that refSeq is more biased towards hg19 minor alleles. It may be worth creating an ethnically normalized refSeq database. As hundreds of thousands of human genomes are sequenced in the future, it is likely that many positions in hg19KIndel may be rendered minor or may lose majority status. However, given that the 1000 Genome project represents carefully-selected ethnically-diverse population, major alleles from this population is likely to remain major; making hg19KIndel a standard reference genome for a long time to come.

As we can see in Table 1 there are some structural variants that are major alleles. Since not all these structural variants were resolved up to a single base level, replacing them is non-trivial. In addition, some of the major structural variants intersected with geneic regions and incorporating them will make the task of generating a compatible annotation for hg19KIndel much more complicated. These will be incorporated in the next version of the release.

## Conclusion

hg19KIndel is the first effort to horizontally improve a human genome assembly to generate a human reference genome that is ethnically normalized. The hg19KIndel offers a reference that eliminates false positive and false negatives stemming from minor alleles in hg19; offers better sensitivity in variant prediction by improving alignment of reads around minor alleles; and provides opportunity to reannotate major disease databases such as COSMIC and ClinVar. While false negatives are missed totally from the analysis of millions of samples in the past using hg19, false positives have a real emotional price to pay in the way of false diagnosis as personalized genome becomes a norm in the future. We strongly recommend use of hg19KIndel for variant calls in the future especially for creating demography-specific SNP databases. We believe that the approach taken here to create hg19KIndel can be used to create demographically normalized reference genomes in the future.

## Methods

### Creation of hg19KIndel

The variants in 1000 Genome database are classified into five categories: a) SNP (Single Nucleotide Polymorphism), b) INDEL (Insertions and Deletions), c) SNP&INDEL (At a particular single position both SNP as well INDELs are present), d) MNP (Multiple Nucleotide Polymorphism) and e) SV (Structural variants such as Copy Number Variation (CNV), large scale deletions, insertions, duplications, inversions etc). For each class of variants, positions having alternate allele frequency > 0.5 were filtered out from the original file such that every class has its own variant call files (vcf) file. In case of multi-allelic sites, the alternate allele having the frequency > 0.5 was selectively extracted and ALT field of the vcf file was updated accordingly. The resulting vcf file generated had only one ALT allele for a given REF allele. All this is done using python scripts developed in-house. In order to make hg19K we used a tool called *FastaAlternateReferenceMaker* from GATK [23]. hg19 was downloaded from ftp site of the Broad Institute which was numerically sorted and was soft-masked, which was later converted to unmasked for further processing. This particular genome version and the vcf file created above (having an alternate AF > 0.5) was given as input to GATK *FastaAlternateReferenceMaker* thereby generating hg19K [7]. The rest of the three variant files representing INDELs, SNP&INDELs and MNP were combined using GATK tools *CombineV*ariants creating a file called *combined.vcf*. This helped in merging duplicate records into one common representation so that there is no ambiguity left when we are trying to replace such variants. Previously generated hg19K and the final combined vcf file was given as input to a python script developed in-house that replaced all the REF allele with ALT allele. Since GATK *FastaAlternateReferenceMaker* can replace only simple INDELs and not the complex substitutions type, an in-house script was written to create hg19KIndel. hg19KIndel was thus generated and thoroughly validated before proceeding to any further steps.

### Variant calling pipeline

Our goal was to do a differential comparison of variants obtained using two versions of genomes including hg19 and hg19KIndel. The idea here is to map a common test dataset to both the genomes while keeping rest of the pipeline same and then compare the differences in the variant calls. Bowtie2 [24] was used to map short reads to the genome. The resultant sam files were converted (into bam), sorted and indexed using SAMtools [25]. Picard tools (http://broadinstitute.github.io/picard/) *MarkDuplicates* was used to remove PCR duplicates. SAMtools *mpileup* and BCFtools [26] *call* was used to actually call the variants. Low quality variants were filtered out using the criteria QUAL> 10 and DP > 3. Since our goal was to compare variants, we had to normalize the variants to bring it to a common representation. *Vcfallelicprimitive* was used to break down complex variants into more simpler forms (SNP and simple INDELs) and *vtnormalize* was used to normalize parsimonious and left-align INDELs.

### Creation of LiftOver

Hg19 and hg19KIndel can effectively be treated as different genome assemblies. hg19KIndel was derived from hg19 by introducing major INDELs thus the genomic coordinates of hg19KIndel do not match to that of hg19. As a result, the variants from hg19KIndel cannot be directly compared with hg19. In order to compare variants called on hg19KIndel with variants called on hg19, the coordinates of variants had to be lifted over from hg19KIndel to hg19. To do this an in-house python script was written, which made use of the *combined.vcf* in order to map every hg19KIndel position to its corresponding hg19 position. The genomics coordinates in the vcf were lifted over; no changes in REF or ALT records were made to make reference allele consistent with the sequence in the target reference hg19. This was kept in mind while comparing the variants and was taken care of further downstream. When there is a deletion in hg19 w.r.t to hg19KIndel, the variant calls at such positions in hg19KIndel could not be lifted over since there was no corresponding position present in hg19. Such variants were classified as an *unmapped* set, which compromised a very miniscule overall percentage (~ 0.15% for whole genome and ~ 0.1% for exome) of the total variants called. Subsequently a chain file hg19KIndeltohg19.over.chain was also generated to convert hg19KIndel coordinates to hg19. This was done by generating a quasi-global-alignment between hg19 and hg19KIndel and finding corresponding syntenic alignment blocks. PyLiftover (https://pypi.org/project/pyliftover/) along with hg19KIndeltohg19.over.chain was used to liftover coordinates. The liftover (converted) files from both the above methods was compared and a concordance of 100% was obtained among them.

### Comparison

VCFtools [27] was used for comparing variants called on different genomes with -*-diff-site* option to compare variants strictly on the basis of position only. This option is critical since the REF and ALT records were not altered during liftover procedure. The output from VCFtools was further processed in order to classify the variants as either common or unique to a particular variant call set. For example, heterozygous sites have their REF and ALT records interchanged when called on different references (having different allele at a particular positions) but essentially, they represent the same variant. Overlapping INDELs around the same vicinity were classified as similar variants. Similarly, some other borderline and ambiguous cases were resolved to classify them into either of the sets. Also, to get a better perspective of changes occurring while using different genomes, numbers for both the classes of variants; SNPs as well as INDELs were separately extracted and compared for final analysis.

### Building gene annotation file and SnpEff database

SnpEff [20] helps in variant annotation and functional prediction. Since the underlying genomes are different, we cannot directly use the existing SnpEff databases in order to annotate variants called by hg19K and hg19KIndel. In order to build a database SnpEff we require: 1) the genome in fasta format and 2) a gene annotation file that describes the gene structure in terms of genomic coordinates like UCSC RefSeq file, GTF, GFF etc. For making SnpEff database for hg19 and hg19K, UCSC refGene table [t*rack: refseq Gene; February 2018; generated by UCSC by aligning the NCBI RefSeq RNAs to hg19*] was downloaded from UCSC table browser. It gives the gene annotation with respect to hg19 genomic coordinates. SnpEff database for hg19K was quite straightforward; since the coordinates of hg19 and hg19K are compatible directly used the hg19 gene annotation file and hg19K genome fasta file. Also, in order to do fair comparisons, the SnpEff database for hg19 was also built inhouse using the hg19 fasta file and hg19 gene annotation file. But in case of hg19KIndel due to incompatibility between coordinates, hg19 gene annotation could no longer be used. This led to the requirement of new gene annotation file which describes gene structure in terms of genomic coordinates of hg19KIndel. Using liftover procedure previously discussed we lifted the entire hg19 gene annotation to hg19KIndel coordinate frame. But lifting just coordinates is not enough. Since the sequence extracted from the genome using the annotation finally represent a functional protein sequence, mismatch of even one base of an exon boundary changes the entire protein being translated. refGene table also provides information about the frame for each exon for each individual transcript. SnpEff uses this frame information for translation of sequences extracted from genome. Consequently, after lifting over coordinates another step of error correction was done to check if the frame information is consistent with hg19 annotation, as well as updating the frame and sometimes the total number of exons in genes that had putative sequencing errors. This error correction was done using an in-house python script. After doing it the genes still having out of frame INDELs that were not be fixed by the above-mentioned procedure were looked at case by case basis and if possible corrected (by own interpretation subjectively). Albeit there were some cases we couldn’t correct and were left as it is. The hg19KIndel gene annotation file created was called as *refGeneKIndel*. To perform sanity check while building SnpEff database we require; a) transcript file: containing the RNA sequences of all transcripts present in the annotation file and b) protein file: containing protein sequences (translated from mRNA sequence) of all transcripts (coding) present in the annotation file. Transcript sequences downloaded from UCSC - *refMrna* file [February 2018] and corresponding protein sequences from RefSeq were given as one of the inputs to SnpEff while building the database. Using the derived gene annotation for hg19KIndel (*refGeneKIndel*) and the hg19KIndel genome fasta file we built a SnpEff database that could be used to understand the functional implications of variants called using hg19KIndel.

## Data Availability

NA12878 whole genome data (ERR194147 ~ 50X depth;ftp.sra.ebi.ac.uk/vol1/fastq/ERR194/ERR194147/). NA12878 exome (NIST7035 – Library 1) was downloaded from GIAB (https://ftp-trace.ncbi.nih.gov/giab/ftp/data/NA12878/Garvan_NA12878_HG001_HiSeq_Exome/). Code availability https://github.com/harsh-shukla/hg19KIndel

## Abbreviations

AF: Allele Frequency
CNV: Copy Number Variation
COSMIC: Catalogue of Somatic Mutations in Cancer
FN: False Negative
FP: False Positive
INDELs: Insertion and Deletions
MNPs: Multiple Nucleotide Polymorphism
SNPs: Single Nucleotide Polymorphism
SV: Structural variant
UCSC: University of California at Santa Cruz
VCF: Variant Calling File
WES: Whole Exome Sequencing
WGS: Whole Genome Sequencing
